# Effects of *Mycobacterium vaccae* vaccine in a mouse model of *tuberculosis: protective action and* differentially expressed genes

**DOI:** 10.1186/s40779-020-00258-4

**Published:** 2020-06-03

**Authors:** Wen-Ping Gong, Yan Liang, Yan-Bo Ling, Jun-Xian Zhang, You-Rong Yang, Lan Wang, Jie Wang, Ying-Chang Shi, Xue-Qiong Wu

**Affiliations:** Army Tuberculosis Prevention and Control Key Laboratory/Beijing Key Laboratory of New Techniques of Tuberculosis Diagnosis and Treatment, Institute for Tuberculosis Research, the 8th Medical Center of Chinese PLA General Hospital, Beijing, 100091 China

**Keywords:** *Mycobacterium tuberculosis*, Immunotherapeutic effect, Immunotherapy, Vaccae vaccine, Differentially expressed genes, Signaling pathway

## Abstract

**Background:**

Tuberculosis is a leading cause of death worldwide. BCG is an effective vaccine, but not widely used in many parts of the world due to a variety of issues. *Mycobacterium vaccae* (*M. vaccae*) is another vaccine used in human subjects to prevent tuberculosis. In the current study, we investigated the potential mechanisms of *M. vaccae* vaccination by determining differentially expressed genes in mice infected with *M. tuberculosis* before and after *M. vaccae* vaccination.

**Methods:**

Three days after exposure to *M. tuberculosis* H37Rv strain (5 × 10^5^ CFU), adult BALB/c mice randomly received either *M. vaccae* vaccine (22.5 μg) or vehicle via intramuscular injection (*n* = 8). Booster immunization was conducted 14 and 28 days after the primary immunization. Differentially expressed genes were identified by microarray followed by standard bioinformatics analysis.

**Results:**

*M. vaccae* vaccination provided protection against *M. tuberculosis* infection (most prominent in the lungs). We identified 2326 upregulated and 2221 downregulated genes in vaccinated mice. These changes could be mapped to a total of 123 signaling pathways (68 upregulated and 55 downregulated). Further analysis pinpointed to the MyD88-dependent TLR signaling pathway and PI3K-Akt signaling pathway as most likely to be functional.

**Conclusions:**

*M. vaccae* vaccine provided good protection in mice against *M. tuberculosis* infection, via a highly complex set of molecular changes. Our findings may provide clue to guide development of more effective vaccine against tuberculosis.

## Background

Since the discovery of *Mycobacterium tuberculosis* by Robert Koch over a century ago [1], human beings have made significant achievements in the fight against tuberculosis (TB). However, with the increase of multidrug-resistant (MDR) strains, human immunodeficiency virus (HIV) co-infection, and lack of effective TB vaccines, TB remains a major threat to human health [2].

Bacillus Calmette–Guérin (BCG), the first vaccine used against TB, is prepared from a strain of the attenuated live *Mycobacterium bovis*. A major limitation of BCG is the variable efficacy across ethnicity and population [2–4]. Vaccae™ vaccine is one of the most promising vaccines against TB. It is a non-cell *Mycobacterium vaccae* vaccine produced by Anhui Zhifei Longcom [5]. Our previous study suggested that it played an important role in improving immunity, promoting phagocytosis, regulating bidirectional immunoreaction, and reducing pathological damage [2]. At present, this vaccine has been given a Chinese new drug certificate and approved by the China Food and Drug Administration (CFDA) for the adjuvant treatment of TB. Currently, a large double-blind Phase III trial has been completed to evaluate the efficacy and safety of the Vaccae™ vaccine in 10,000 cases whose skin tests of PPD (purified protein derivative) were strongly positive in Guangxi province in China [6], and the results have not yet been published.

*M. vaccae* is a nonpathogenic species of the Mycobacteriaceae family and belongs to the same genus as *M. tuberculosis*. This bacterium contains many protective antigens with immunomodulating effects [2, 7]. Previous studies in animal models have demonstrated that *M. vaccae* vaccine had a good immunotherapeutic effect by stimulating T lymphocytes producing high-level cytokines such as interferon-gamma (IFN-γ) [8], interleukin 12 (IL-12) [9], IL-4 delta 2 [10], and tumor necrosis factor-alpha (TNF-α) [11]. Furthermore, this vaccine also has been used as an immunotherapeutic adjunct to treat TB [12–14], MDR-TB [15], surgery-elicited neuroinflammation and cognitive dysfunction [16], metastatic malignant melanoma [17], and neuroimmune processes [18].

Although the potential mechanisms of *M. vaccae* vaccine immunotherapy have been studied from the immunological and proteomic levels [19–21], the molecular mechanism of the immunotherapeutic effect of this vaccine is still unclear. Herein, we assessed the immunotherapeutic effect of the *M. vaccae* vaccine in mouse animal model and identified the differential expression (DE) genes of mice before and after *M. vaccae* vaccine treatment for the first time by using DNA (deoxyribonucleic acid) microarray. Based on these data, we hope to identify possible target molecules and signaling pathways of *M. vaccae* vaccine, which will give a new perspective for the molecular mechanism of its immunotherapy.

## Methods

### Mice and ethics statement

Female BALB/c mice (6–8 weeks of age) were purchased from the Institute of Military Medicine, Academy of Military Sciences of Chinese PLA (People’s Liberation Army) (Beijing, China). Experimental protocol was approved by the Animal Ethical Committee of the 8th Medical Center of Chinese PLA General Hospital, and conducted in compliance with the Experimental Animal Regulation Ordinances of the China National Science and Technology Commission.

### Mycobacterial strains and *M. vaccae* vaccine

*Mycobacterium tuberculosis* (H37Rv strain) was cultured and purified as previously described [22, 23]. *M. vaccae* vaccine (Vaccae™) was purchased from Anhui Zhifei Longcom Co., Ltd. (Anhui, China).

### Immunization and challenge

**Fig. 1:**
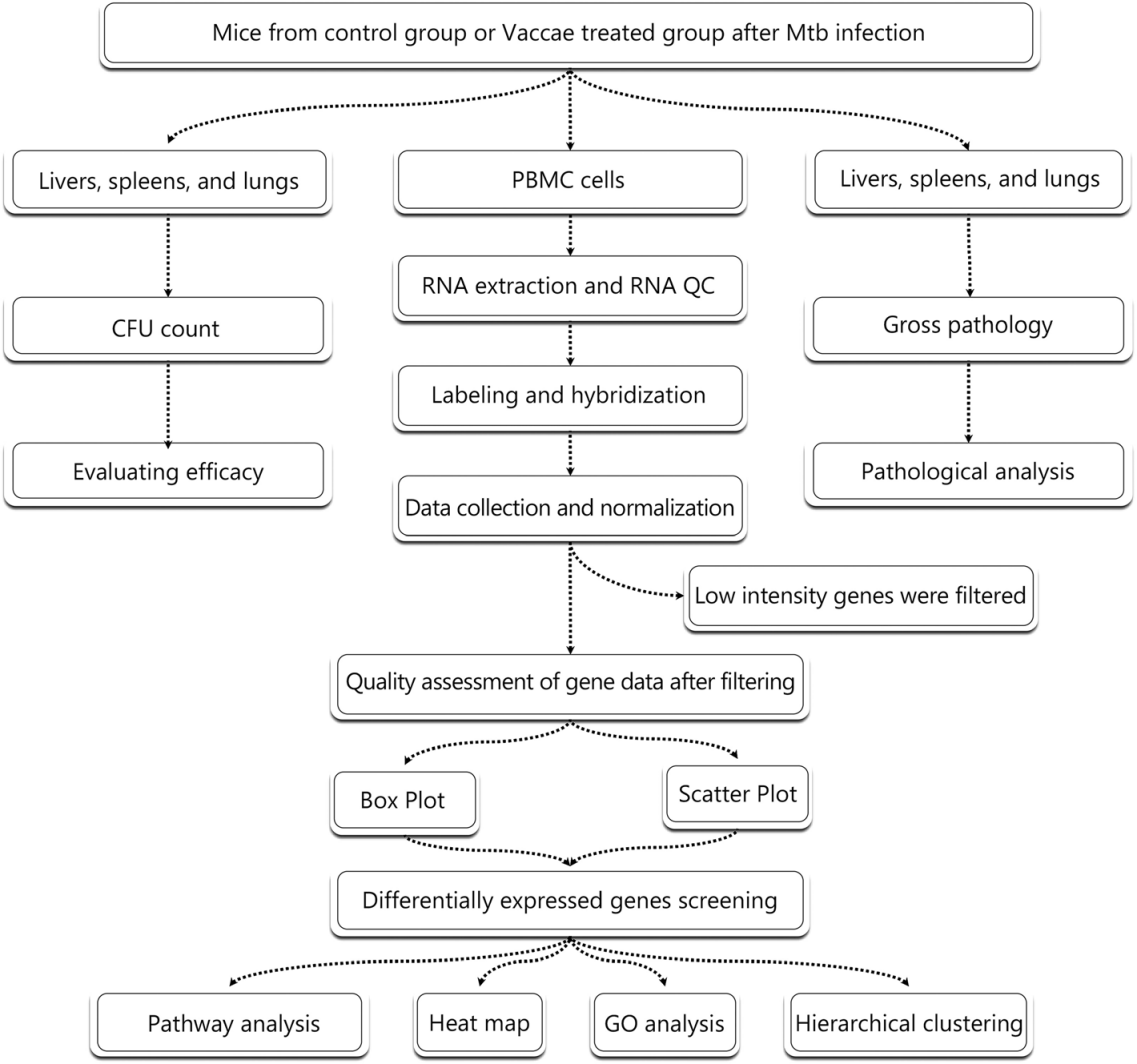
The flow chart of all experiments. Eight mice of *M. vaccae* group or control group were challenged with *M. tuberculosis* H37Rv strain. After 3 times immunization, mice were sacrificed and their lungs, livers, and spleens were collected to efficacy evaluation and pathological observation. The PBMCs of 3 mice of each group were isolated to extract total RNA. Hierarchical Clustering was performed to show the distinguishable gene expression profiling between samples. DE genes with statistical significance were identified through Volcano Plot filtering. Finally, pathway analysis and GO Analysis were applied to determine the roles of these DE genes played in these biological pathways or GO terms. GO, Gene Ontology; PBMCs, peripheral blood mononuclear cells; CFUs, colony formation units

**Fig. 2:**
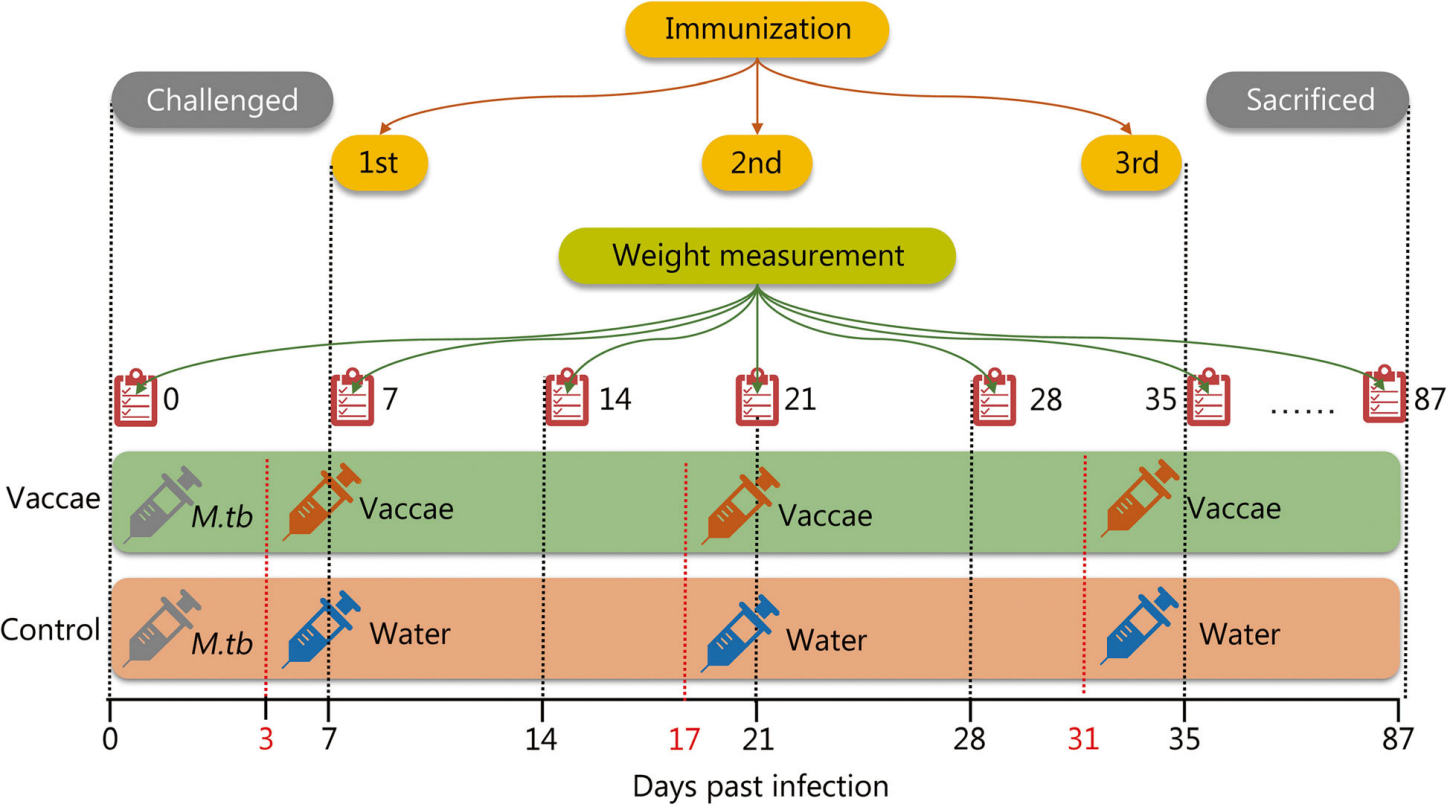
The schedule of immunotherapy evaluation. Each mouse in the control group (showed as an orange belt) and *M. vaccae* group (showed as a green belt) was challenged with *M. tuberculosis* H37Rv strain at day 0. Three days after challenge, mice were immunized intramuscularly 3 times at 2-week intervals with water (orange belt) and *M. vaccae* vaccine (green belt), respectively. Eighty-seven days after challenge, each mouse was sacrificed and their organs were collected for protective evaluation and histopathological analysis. Additionally, the weight of each mouse was measured per week (showed as a red notebook)

General experimental design is shown in Fig. 1. The schedule of immunization and challenge is shown in Fig. 2. Mice received 5 × 10^5^ colony formation units (CFUs) of *M. tuberculosis* H37Rv strain via the caudal vein. Three days later, mice were randomly divided to receive intramuscular injection of either *M. vaccae* vaccine (22.5 μg in 100-μl distilled water) or vehicle (*n* = 8). Booster immunization was conducted 14 and 28 days after the primary immunization.

### Infection severity assessment

Mouse body weight was measured once per week. Eight weeks after the last immunization, the mice per group were killed and the lungs, liver, and spleen were collected for gross pathological observation, histopathological examination, and CFU counting. Firstly, the organ coefficients were evaluated by the ratio of organ weight to body weight, the average areas of lesions in the liver, the number of the tubercular nodules in the lung, and the size of spleen were observed following the standards listed in Table 1. The spleen and the left lobe of lung were homogenized in 3-ml saline, serially diluted (10-fold in each step), inoculated in duplicate on Lowenstein-Jensen medium plate (100 μl) and cultured at 37 °C for 4 weeks. Colonies on the medium were counted and the results are showed as CFUs per organ. The right lung was fixed in 10% (vol/vol) formalin overnight and embedded in paraffin. Sections (3 μm) thickness were stained with hematoxylin and eosin (H&E) for histopathological examination as previously described [22–28].

**Table 1:** The standard of identifying gross pathological lesion indexes of organs by pathological observation

Organs	Lesion indexes			
	–	1+	2+	3+
Lung	Without TB nodules and caseous necrosis	The number of TB nodules ≤10 or the area of caseous necrosis up to 20%	The number of TB nodules ≥10 or the area of caseous necrosis up to 40%	The number of TB nodules ≥20 or the area of caseous necrosis up to 40%
Liver	Normal size	Slight swell ^a^	Moderate swell ^b^	Severe swell ^c^
Spleen	Normal size	Slight swell ^a^	Moderate swell ^b^	Severe swell ^c^

Note: Relative to normal liver and spleen, ^a^ swell was less than 20%; ^b^ swell was more than 20% and less than 40%; ^c^ swell was more than 40%

### PBMCs isolation and total RNA extraction

On days 87 after challenge, 3 mice of each group were sacrificed. PBMCs (peripheral blood mononuclear cells) were prepared using a Mouse PBMCs Isolation Kit (TBDscience, Tianjin, China). Total RNA was extracted using a kit from Solarbio Life Science (Beijing, China). The integrity of RNA was assessed by electrophoresis on a denaturing agarose gel. Sharp 28S and 18S rRNA bands at a ratio of 2:1 are used as the hallmark for intact RNA.

### Sample RNA purity and concentration

The NanoDrop ND-1000 was used to measure RNA concentration (OD_260_), protein contamination (ratio of OD_260_/OD_280_) and organic compound impureness (ratio OD_260_/OD_230_). The OD_260_/OD_280_ ratio should be > 1.8.

### DNA microarray

DNA microarray experiment was conducted using a Mouse 4x44K Gene Expression Array (Agilent) with 39,000+ mouse genes and transcripts, all with public domain annotations.

### RNA labeling and array hybridization

Sample labeling and array hybridization were conducted according to the Agilent One-Color Microarray-Based Gene Expression Analysis protocol (Agilent Technology). Briefly, total RNA from each sample was amplified and labeled with Cy3-UTP. Labeled cRNAs were purified by RNeasy Mini Kit (Qiagen), and NanoDrop ND-1000 was used to measure the concentration and specific activity of the labeled cRNAs (pmol Cy3/μg cRNA). One microgram of each labeled cRNA was fragmented by adding 2.2-μl 25 × fragmentation buffer and 11-μl 10 × blocking agents, heated at 60 °C for 30 min, and diluted by adding 55- μl 2 × GE hybridization buffer. Then, 100 μl of hybridization solution was added into the gasket slide and assembled to the gene expression microarray slide. The slides were incubated for 17 h at 65 °C in an Agilent Hybridization Oven. The hybridized arrays were washed, fixed and scanned using the Agilent DNA Microarray Scanner (part number G2505C).

### Data analysis

Microarray images were analyzed using Agilent Feature Extraction software (version 11.0.1.1). Quantile normalization and subsequent data processing were performed using GeneSpring GX v11.5.1 software package (Agilent Technologies, USA). DE genes were identified through volcano plot filtering. Hierarchical clustering was performed using the Agilent GeneSpring GX software (version 11.5.1). GO analysis and KEGG (Kyoto Encyclopedia of Genes and Genomes) pathway analysis were performed using a standard enrichment computation method.

### Statistical analysis

Statistical analyses were conducted using SAS (version 9.1, SAS Institute, Cary, NC). The sample size was estimated according to our previous studies [24–27]. The results of *M. vaccae* protective experiments, gross pathological observation, histopathological examination, and CFU count were compared with Student’s *t*-test or Wilcoxon Two-Sample test according to data normality and homogeneity of variances. Differential expression was defined as fold-change ≥2. *P* < 0.05 was considered statistically significant.

## Results

### Efficacy of the vaccine

**Fig. 3:**
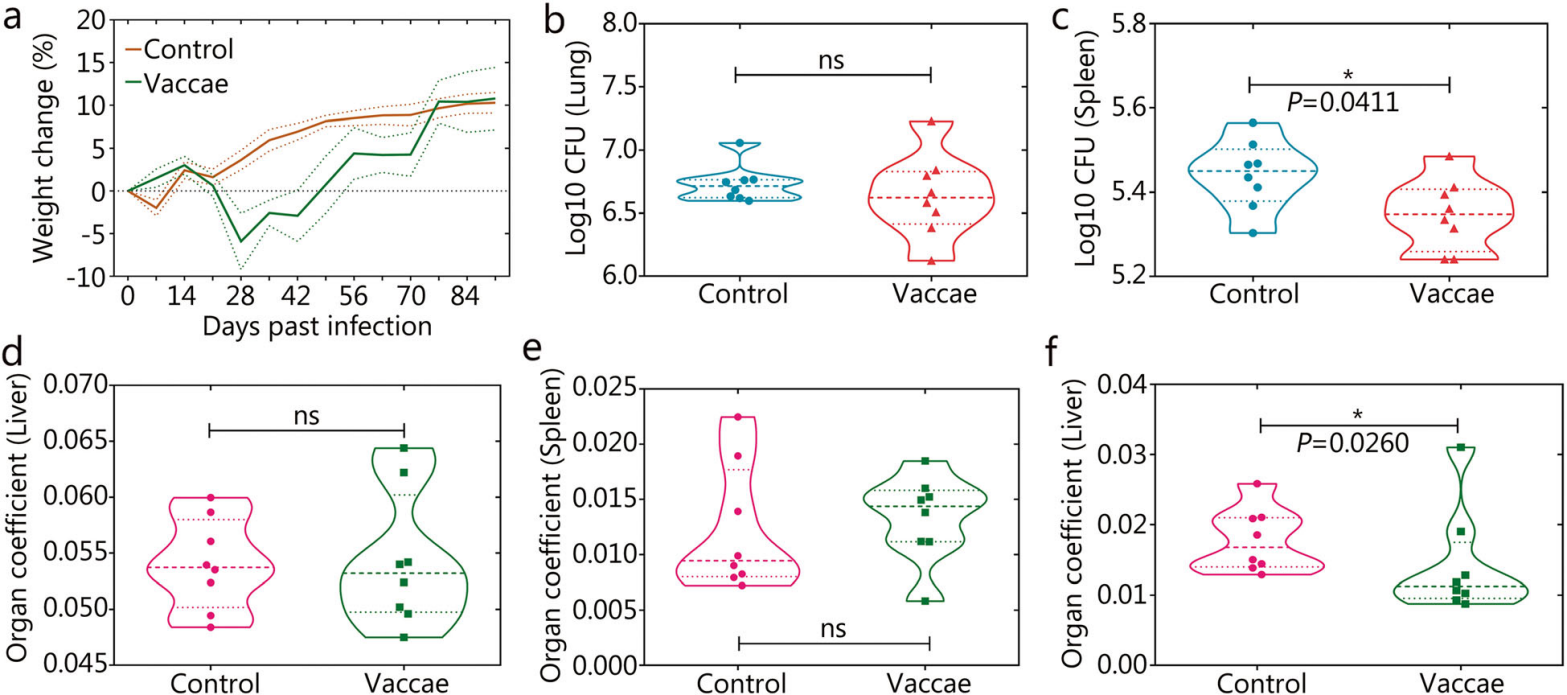
Immunotherapeutic efficacy of *M. vaccae* vaccine. After the challenge, the weight change of each mouse was measured weekly (**a**, the error bar is represented by dotted lines). Ninety-one days after challenge, all of the mice were killed and their left lobe of the lung **b** and spleen **c** was collected for CFU counting. Additionally, organ coefficient of the lung **d**, spleen **e**, or liver **f** was performed. All data are presented as means + S.E.M. (*n* = 8). Differences were considered statistically significant at *P* < 0.05. *, *P* < 0.05; ns. Not significant

With the exception of temporary reduction in the first week, body weight in mice receiving the vehicle increased over the entire experimental period as expected (Fig. 3a). In mice receiving the *M. vaccae* vaccine, body weight started to decrease on day 14 day, reached a nadir on day 28, and then increased gradually back to the control level on day 77 (Fig. 3a).

In comparison to the control, there was a statistically non-significant trend for decreased CFUs in the lungs in the *M. vaccae* group (Fig. 3b). The CFUs in the spleen was lower in the *M. vaccae* group (*P* = 0.041 vs. control, Fig. 3c). In comparison to the control, mice in the *M. vaccae* group had similar organ coefficient of the liver (Fig. 3d) and spleen (Fig. 3e), but significantly lower organ coefficient of the lungs (*P* = 0.026, Fig. 3f).

### Histopathological and gross pathological analyses

**Fig. 4:**
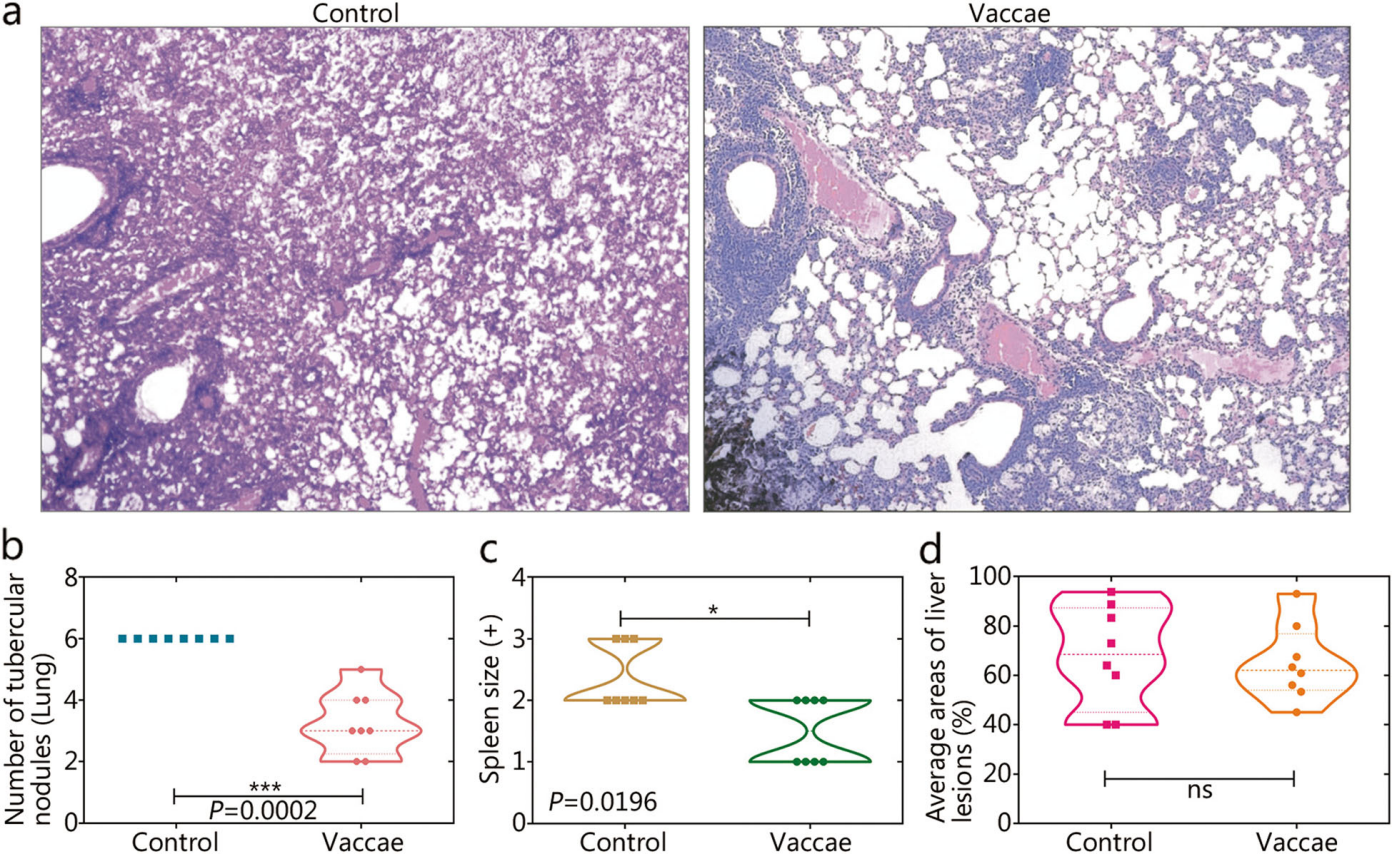
Gross pathology and histopatological analysis. The right lobe of lungs collected from the mice in the control group (**a**, left) and *M. vaccae* group (**a**, right) were used to undergo histopathological examination (H&E). The gross pathology of organs was also observed, including the number of the tubercular nodules in the lung **b**, spleen size average **c**, and areas of lesions in the liver **d**. Original magnification times: ×100. All data are presented as means + S.E.M. (*n* = 8). Differences were considered statistically significant at *P* < 0.05. *, *P* < 0.05; ***, *P* < 0.001; ns. Not significant

The structure of alveoli was damaged severely in the control group (Fig. 4a). Inflammatory cell infiltration of the lungs was apparent in the *M. vaccae* group, but the alveolar wall was intact, with no thickening. Gross pathological analysis showed fewer tubercular nodules in the lungs (*P* = 0.0002, Fig. 4b) and decreased spleen size (*P* = 0.0196, Fig. 4c) in the *M. vaccae* group. The average area of the lesions in the liver did not differ significantly between the two groups (Fig. 4d).

### DE genes

**Fig. 5:**
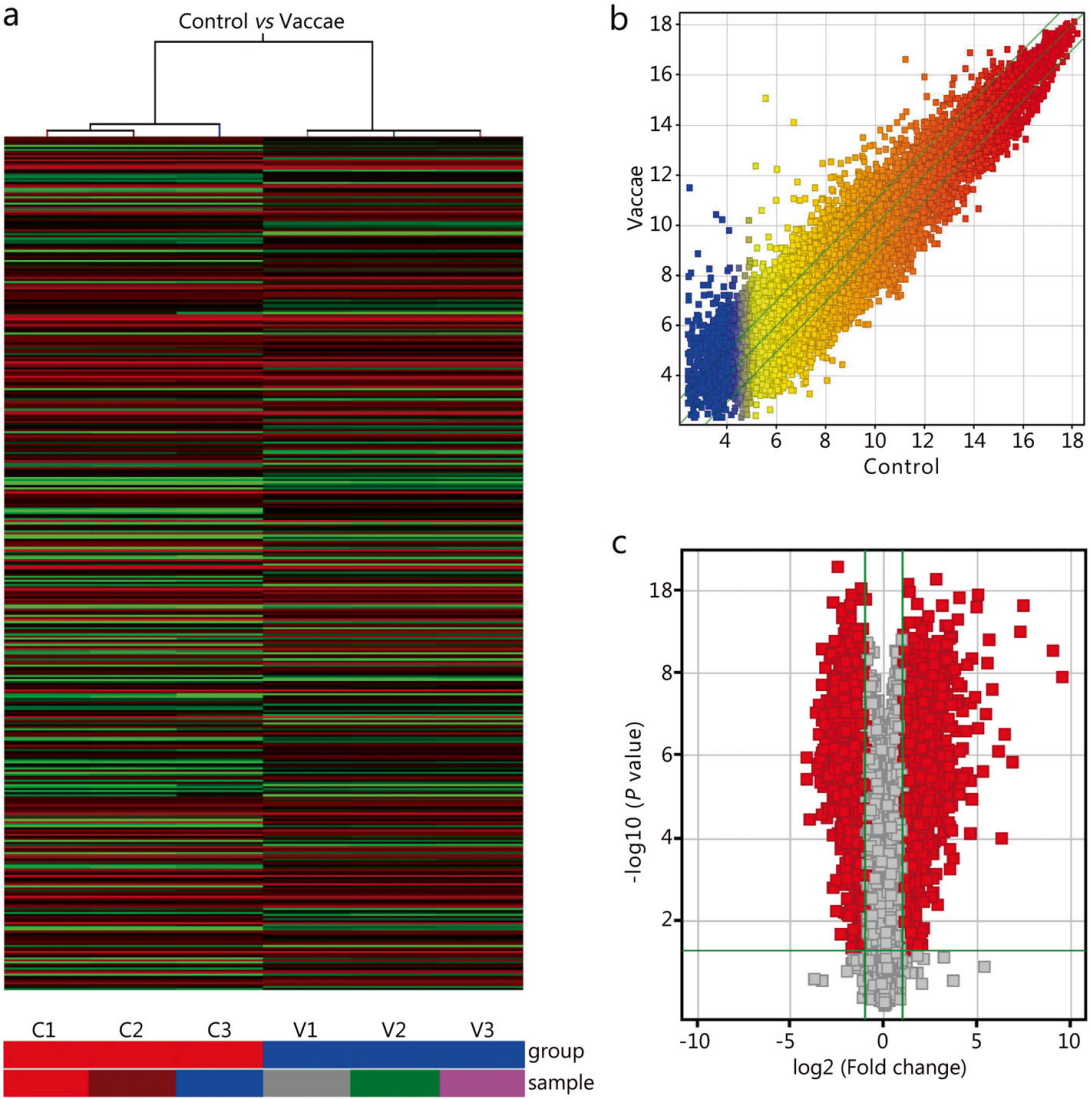
Heat map, hierarchical clustering presentation, scatter plot, and volcano plot of the expression profile of genes in control and *M. vaccae* groups. **a**. Heat map and hierarchical clustering showed the relationships among gene expression patterns of samples (*n* = 3). Red indicates high relative expression, and green indicates low relative expression. **b**. Scatter plot, the values of X and Y axes in the scatter plot are the normalized signal values of the samples (log2 scaled) or the averaged normalized signal values of the groups (log2 scaled). The green lines are Fold Change Lines (the default fold change value given is 2.0). The genes above the top green line and below the bottom green line indicated more than a 2-fold change of genes between two samples or groups (*n* = 3). **c**. DE genes with statistical significance were identified through the volcano plot, and the red diamonds represented DE genes with fold change ≥2.0, *P* ≤ 0.05 (*n* = 3). C1 – C3, sample 1 to sample 3 in control group; V1-V3, sample 1 to sample 3 in Vaccae group

Agilent Mouse 4x44K Gene Expression Microarrays v2 was used to identify DE genes. The array image of each sample was obtained (Fig. S1), and the intensity data was extracted. After quantile normalization of the raw data, genes listed in Table S1 were chosen for DE gene screening. Differential gene expression is shown using a heat map, hierarchical cluster (Fig. 5a) and scatter plot (Fig. 5b). The DE genes were screened with volcano plot (Fig. 5c). We identified 2326 upregulated genes and 2221 downregulated genes in the *M. vaccae* group. The top 20 upregulated genes were *Retnlg*, *Tiprl*, *Gyg*, *Ptgs2*, *Zfp281*, *Gbp2*, *Cxcl2*, *Ear6*, *Ighv1–77*, *Slpi*, *Azin1*, *S100a8*, *Ccrl2*, *Igj*, *Tnf*, *Prkcd*, *Marcksl1*, *Prss34*, *Cct6a*, and *Il1a* (Table 2). The top 20 downregulated genes were *Afp*, *Pcdhga9*, *Cdc42ep5*, *Hrsp12*, *Rnasek*, *Nprl3*, *4932443I19Rik*, *Ly6g6c*, *Kdr*, *2810416G20Rik*, *Tubb2a*, *Triqk*, *Slc6a16*, *Cxx1c*, *Fez2*, *1810058I24Rik*, *Egfbp2*, *Efna5*, *Cd151*, and *2210013O21Rik* (Table 2). More detailed information (all DE genes) are shown in Table S2.

**Table 2:** Top 20 DE genes between control and *M. vaccae* groups

Gene name	*P*-value fold change and regulation				GenBank accession	Relationship with TB
	*P*-value ^a^	FDR ^b^	FCAbsolute ^c^	Regulation		
Retnlg	5.2646E-11	3.00721E-07	6.762246	Up	NM_181596	Unknown
Tiprl	6.7691E-11	3.00721E-07	2.3434467	Up	NM_145513	Unknown
Gyg	1.0686E-10	3.00721E-07	2.590874	Up	NM_013755	Unknown
Ptgs2	1.1782E-10	3.00721E-07	33.16254	Up	NM_011198	Decreased transcription of PTGS2 was beneficial to the survival of *M. tuberculosis*
Zfp281	1.9767E-10	3.00721E-07	3.3773346	Up	NM_177643	Unknown
Gbp2	2.162E-10	3.00721E-07	8.493201	Up	NM_010260	One of prominent hubs in a highly active common core in TB
Cxcl2	2.2714E-10	3.00721E-07	176.0849	Up	NM_009140	Blocking of CXCL2 can significantly reduce the M. tuberculosis-induced IL-1β production
Ear6	2.4307E-10	3.00721E-07	30.299973	Up	NM_053111	Unknown
Ighv1–77	4.1282E-10	4.21673E-07	4.998427	Up	AF045501	Unknown
Slpi	5.6974E-10	4.97498E-07	3.1604574	Up	NM_011414	Exposure of murine peritoneal macrophages to *M. tuberculosis* led to an increase in SLPI secretion accelerating both the phagocytosis and killing of the pathogen
Azin1	5.9447E-10	4.97498E-07	2.5270393	Up	NM_018745	Unknown
S100a8	7.0447E-10	4.97498E-07	9.958652	Up	NM_013650	A major pathologic role for S100A8/A9 proteins in decreasing lung tissue damage without impacting protective immunity against TB
Ccrl2	7.0963E-10	4.97498E-07	10.672567	Up	NM_017466	Unknown
Igj	8.5416E-10	5.25022E-07	4.127778	Up	NM_152839	Unknown
Tnf	9.694E-10	5.66345E-07	156.13841	Up	NM_013693	TNF-α has a prominent role in defense and pathological responses to TB and its production in TB patients was higher than that in the control group
Prkcd	1.1807E-09	6.28255E-07	2.054293	Up	NM_011103	Unknown
Marcksl1	1.2759E-09	6.28255E-07	11.7962	Up	NM_010807	Unknown
Prss34	1.3143E-09	6.28255E-07	11.188594	Up	NM_178372	Unknown
Cct6a	1.4218E-09	6.40172E-07	2.9756305	Up	NM_009838	Unknown
Il1a	1.5183E-09	6.40172E-07	48.50243	Up	NM_010554	The expression of the IL1A gene was increased in both the TB-infected and the healthy cattle to *M. bovis* stimulation
Afp	2.4759E-11	3.00721E-07	5.7038527	Down	NM_007423	Only a few literatures reported that AFP was normal in TB patients, but increased significantly in TB patients with hepatocellular carcinoma
Pcdhga9	8.4144E-11	3.00721E-07	2.3555577	Down	NM_033592	Unknown
Cdc42ep5	1.2007E-10	3.00721E-07	3.3431478	Down	NM_021454	Unknown
Hrsp12	1.5283E-10	3.00721E-07	2.9907024	Down	NM_008287	Unknown
Rnasek	1.5763E-10	3.00721E-07	2.0300574	Down	NM_173742	Unknown
Nprl3	1.6254E-10	3.00721E-07	2.5463195	Down	NM_001284359	Unknown
4932443I19Rik	1.9509E-10	3.00721E-07	6.6056542	Down	NM_001101519	Unknown
Ly6g6c	2.3228E-10	3.00721E-07	3.6154532	Down	NM_023463	Unknown
Kdr	2.6218E-10	3.06341E-07	4.521906	Down	NM_010612	Unknown
2810416G20Rik	3.0664E-10	3.39439E-07	4.7047515	Down	XM_003945668	Unknown
Tubb2a	4.3967E-10	4.21673E-07	3.6115813	Down	NM_009450	Unknown
Triqk	4.4108E-10	4.21673E-07	4.918867	Down	NM_173746	Unknown
Slc6a16	6.2508E-10	4.97498E-07	4.1382565	Down	XM_355900	Unknown
Cxx1c	8.1855E-10	5.25022E-07	3.0826645	Down	NM_028375	Unknown
Fez2	8.6019E-10	5.25022E-07	2.6090815	Down	NM_001285940	Unknown
1810058I24Rik	8.7371E-10	5.25022E-07	4.307958	Down	NR_027875	Unknown
Egfbp2	1.0983E-09	6.07878E-07	4.641702	Down	NM_010115	Unknown
Efna5	1.2336E-09	6.28255E-07	2.744601	Down	NM_207654	Unknown
Cd151	1.2898E-09	6.28255E-07	3.2171504	Down	NM_009842	CD9^High^ classical monocytes expressed higher levels of tetraspanin CD151 compared to CD9^Low^ classical monocytes
2210013O21Rik	1.4572E-09	6.40172E-07	3.4838674	Down	NM_027327	Unknown

a, *P*-value calculated from t-test; b, FDR calculated from Benjamini Hochberg FDR; c, FCAbsolute, the absolute ratio (no log scale) of normalized intensities between two groups. Notes: mRNA with expression fold change > 2 and with FDR adjusted *P*-value < 0.05 was considered statistically significant. Here we show the expression fold change > 10

### GO analysis

GO analysis showed that, in comparison with the control group, the upregulated genes involve 1672 terms in biological process (BP, *P* < 0.05, Table S3 BP sheet), 137 terms in cellular component (CC, *P* < 0.05, Table S3 CC sheet), and 231 terms in molecular function (MF, *P* < 0.05, Table S3 MF sheet). The downregulated genes involved 1080 terms in BP (*P* < 0.05, Table S4 BP sheet), 134 terms in CC (*P* < 0.05, Table S4 CC sheet), and 195 terms in MF (*P* < 0.05, Table S4 MF sheet).

**Fig. 6:**
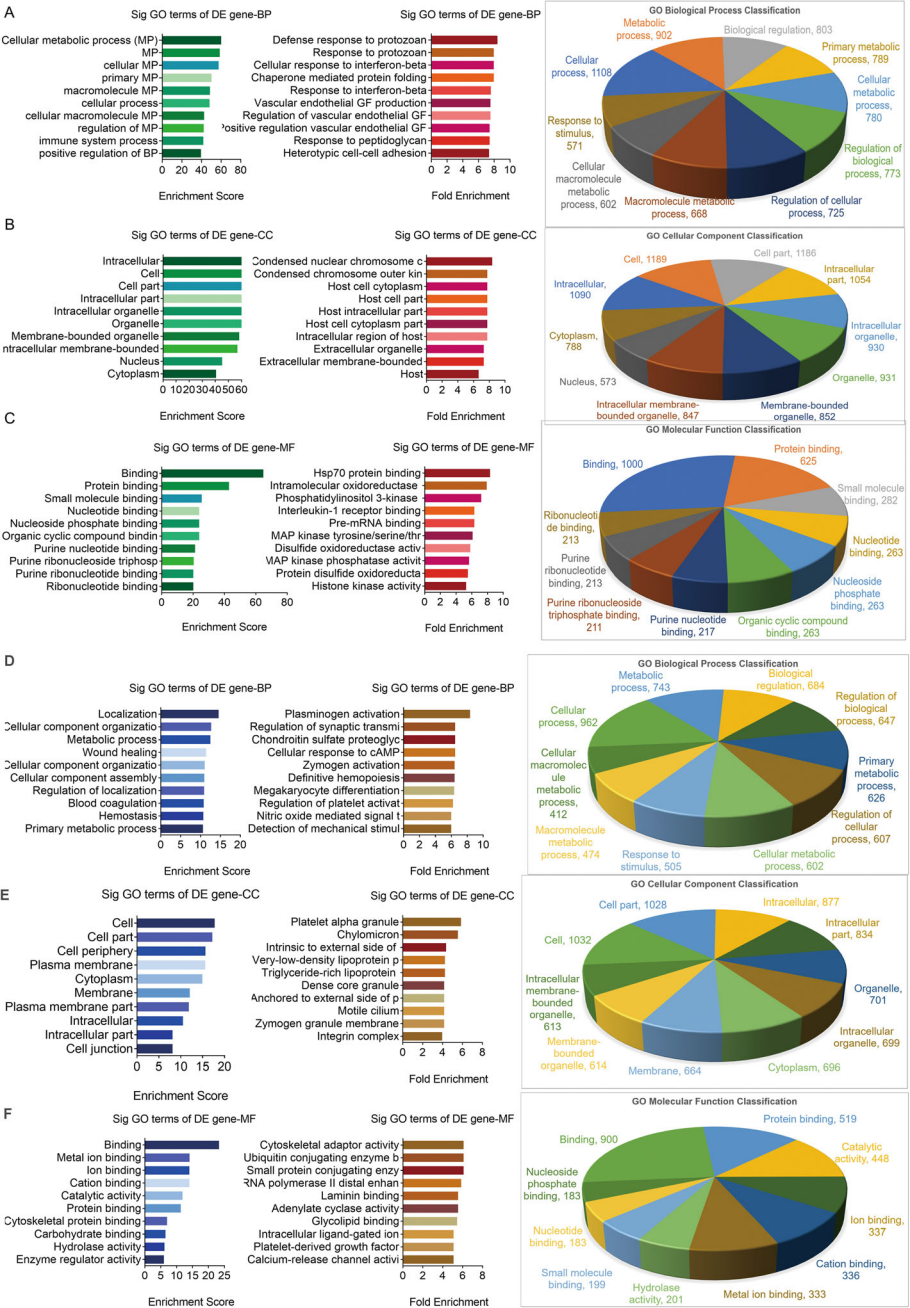
GO analysis of DE genes between two groups. Significant GO terms of TOP 10 upregulated DE genes involved in biological process **a**, cellular component **b**, and molecular function **c** or that of TOP 10 downregulated DE genes involved in biological process **d**, cellular component **e**, and molecular function **f** were identified, respectively. Left lane, Enrichment Score; Middle lane, Fold Enrichment; Right lane, Classification. BP, biological process; CC, cellular component; MF, molecular function. The *P*-value denotes the significance of GO Term enrichment in the DE gene list. The less the *P*-value is, the more significant of the GO Term is (*P* ≤ 0.05 is recommended). DE, differential expression; GO, Gene Ontology; BP, biological process; CC, cellular component; MF, molecular function

The top 10 GO terms of the upregulated genes sorted by enrichment score (left lane in Fig. 6), fold enrichment (middle lane in Fig. 6), and classification (right lane in Fig. 6) in BP, CC, and MF are shown in Fig. 6a, Fig. 6b, and Fig. 6c, respectively. The top 10 GO terms of the downregulated genes are showed in Fig. 6d/E/F. Briefly, the upregulated genes in the *M. vaccae* group are mainly related to metabolic process, cellular metabolic process, primary metabolic process, intracellular, and binding. The downregulated genes are mainly associated with localization, cellular component organization, metabolic process, cell part, cell periphery, and binding.

### Pathway analysis

**Fig. 7:**
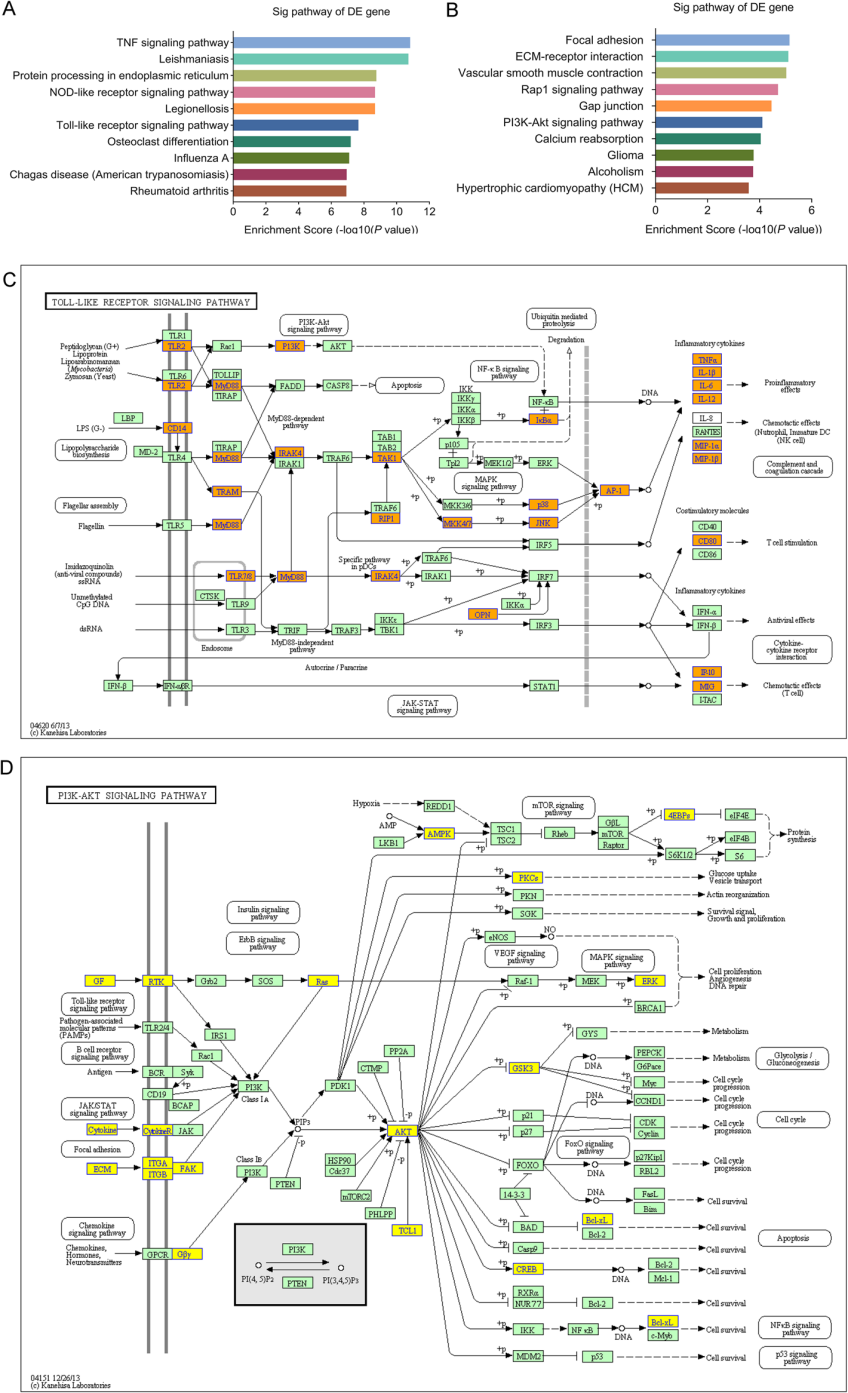
Pathway bar plot explanation and pathway map explanation. The bar plot showed the top 10 enrichment score [−log10 (*P*-value)] value of the significantly upregulated pathways **a** and downregulated pathways **b**. The significantly upregulated signaling pathway **c** and downregulated pathway **d** mostly associated with *M. vaccae* vaccine treatment were selected to show here. Yellow marked nodes are associated with downregulated genes, orange marked nodes are associated with upregulated or only whole dataset genes, green nodes have no significance

KEGG analysis showed that, in comparison with the control group, 68 pathways were unregulated in the *M. vaccae* group (Table S5); the top 10 were mmu04668- tumor necrosis factor (TNF) signaling pathway, mmu05140 Leishmaniasis, mmu04141 protein processing in endoplasmic reticulum, mmu04621 nucleotide-binding oligomerization domain (NOD)-like receptor signaling pathway, mmu05134 Legionellosis, mmu04620 Toll-like receptor (TLR) signaling pathway, mmu04380 osteoclast differentiation, mmu05164 Influenza A, mmu05142 Chagas disease (American trypanosomiasis), and mmu05323 rheumatoid arthritis (Fig. 7a). There were 55 down regulated pathways in the *M. vaccae* group (Table S6); the top 10 were mmu04510 focal adhesion, mmu04512 extracellular matrix (ECM)-receptor interaction, mmu04270 vascular smooth muscle contraction, mmu04015 Rap1 signaling pathway, mmu04540 gap junction, mmu04151 PI3K (phosphatidylinositol-4,5-bisphosphate 3-kinase)-Akt (protein kinase B) signaling pathway, mmu04961 endocrine and other factor-regulated calcium reabsorption, mmu05214-glioma, mmu05034-alcoholism, and mmu05410-hypertrophic cardiomyopathy (Fig. 7b). The upregulated and downregulated pathway mostly associated with *M. vaccae* vaccine was MyD88-dependent TLR signaling pathway (Fig. 7c, *P* = 2.193097 × 10^− 8^) and PI3K-Akt signaling pathway (Fig. 7d, *P* = 7.834627 × 10^− 5^), respectively.

**Fig. 8:**
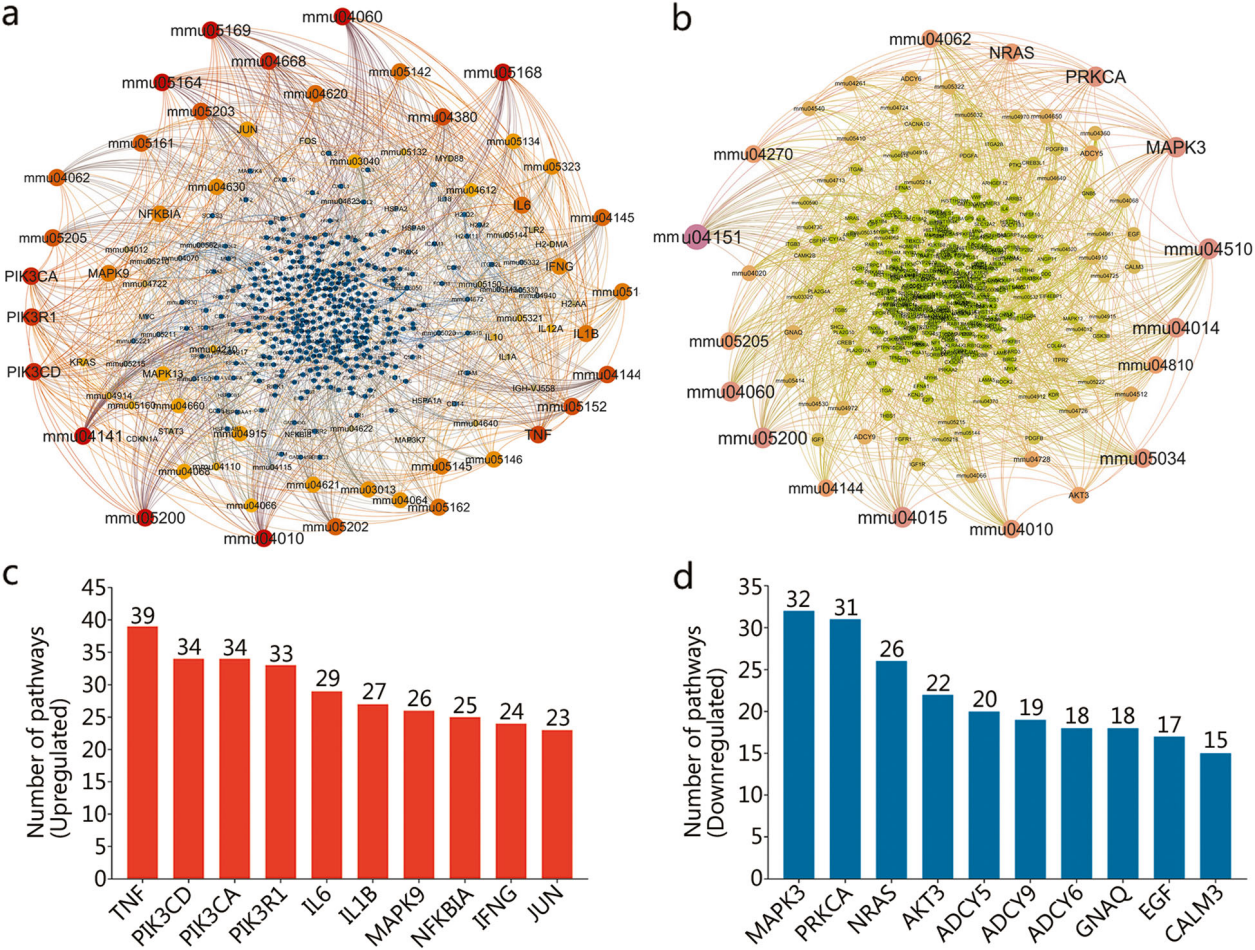
Network map of DE genes associated with upregulated or downregulated pathways. The network map of DE genes associated with upregulated **a** or downregulated **b** pathways was made by Gehpi software. The pathways were showed as pathway ID such as mmu05168. The color and diameter of each circle represent the degree of enrichment of the genes or pathways, the larger diameter, and redder or pinker color indicate the more upregulated or downregulated pathways the gene involved. In addition, the top 10 genes of upregulated **c** or downregulated **d** pathways were also identified

The relationship among DE genes associated with upregulated pathways (Fig. 8a) and downregulated pathways (Fig. 8b) were determined by using Gehpi software. Subsequently, we analyzed the number of upregulated or downregulated pathways involved in a DE gene. The top 10 DE genes associated with upregulated pathways were *TNF*, *Pik3cd*, *Pik3ca*, *Pik3r1*, *Il6*, *Il1b*, *Mapk9*, *Nfkbia*, *Ifng*, and *Jun* (Fig. 8c). The top 10 DE genes associated with downregulated pathways were *Mapk3*, *Prkca*, *Nras*, *Akt3*, *Adcy5*, *Adcy9*, *Adcy6*, *Gnaq*, *Egf*, and *Calm3* (Fig. 8d).

## Discussion

The current study showed that the *M. vaccae* vaccine could decrease the CFUs of *M. tuberculosis* in mice. Such effect was most robust in the spleen, and statistically significant in the lungs. The organ coefficient of the lungs was decreased. The vaccination attenuated the pulmonary lesion and splenomegaly. These results are generally consistent with the lower *M. tuberculosis* CFUs, pathological change index, and organ weight index in previous studies [20, 29], and indicated that *M. vaccae* vaccine had a significant immunotherapeutic effect on TB.

Previous studies suggested that the effects of *M. vaccae* vaccine immunotherapy mainly depend on enhanced recall IFN-γ responses [8], CD3^+^CD4^+^ T cells, IFN-γ^+^CD4^+^ T cells, natural killer (NK) cells, and reduced IL-4^+^CD4^+^ T cells [29]. However, a systematic review of clinical trials conducted suggested no benefit of *M. vaccae* vaccine immunotherapy [30]. One study showed that smooth type of *M. vaccae* could interfere with the production of helper T lymphocytes-1 (Th-1) cytokines, and rough type of *M. vaccae* could induce the production of Th-1 cytokines [31] by splenocytes, suggesting that the different colonial morphology (smooth type or rough type) of *M. vaccae* might affect the immunomodulatory effects of *M. vaccae* preparations. Such discrepancy could have contributed to varying results across different vaccines made with *M. vaccae* in clinical trials.

We speculated that there are significant differences in gene expression profiles before and after *M. vaccae* vaccine treatment, and identifying these changes could help to understand the regulatory mechanism of the *M. vaccae* vaccine. We identified 2326 upregulated genes and 2221 downregulated genes in *M. vaccae* group, suggesting that *M. vaccae* vaccine induce more complex and specific gene regulation activities in individuals infected with *M. tuberculosis*. The top 1 upregulated gene was *Retnlg* (also known as *Xcp1*, *Fizz3*, and *Relmg*), which encodes the resistin-like gamma protein (alternative names, RELM-γ or XCP1). This protein was first identified as a novel member of the resistin-like molecule/found in inflammatory zone (RELM/FIZZ) family in mice and rats [32]. A subsequent study showed marked increase of *Retnlg* expression in spontaneously hypertensive hyperlipidemic rats [33], suggesting that RELM-γ has cytokine-like effect and plays a role in promyelocytic differentiation [32, 34]. In addition to RELM-γ, several other upregulated genes identified in our study have been previously reported to be associated with TB. For example, decreased transcription of PTGS2 has been shown to confer a survival benefit to *M. tuberculosis* [35]. GBP2 is one of the prominent hubs in a highly active common core in TB [36]. Blocking CXCL2 could reduce the *M. tuberculosis*-induced IL-1β production [37, 38]. Exposure of murine peritoneal macrophages to *M. tuberculosis* increases SLPI secretion and accelerates both the phagocytosis and killing of the pathogen [39–41], possibly by interacting with S100A8/A9 proteins to decrease lung tissue damage without affecting protective immunity against TB [42]. TNF-α has a prominent role in defense and pathological responses to TB and its production in TB patients has been shown to be increased by the *M. vaccae* vaccine [43–45]. Expression of the *IL1A* gene is increased in both the TB-infected and the healthy cattle to *M. bovis* stimulation [46].

The top 1 downregulated gene was *Afp* encoding alpha-fetoprotein (AFP). AFP is a shuttle protein that transports nutrients to embryonic cells through receptor-mediated endocytosis and converts drugs into AFP-positive bone marrow-derived inhibitors in adults. Previous studies have implicated AFP in the regulation of cell growth, differentiation, apoptosis, angiogenesis, and immune regulation [47]. A few previous studies reported normal AFP in TB patients, but increased AFP in TB patients with hepatocellular carcinoma [48, 49]. Monocytes can undergo homotypic fusion to produce different types of multinucleated giant cells in response to *M. tuberculosis* infection. In comparison to CD9^Low^ classical monocytes, CD9^High^ classical monocytes expressed higher levels of tetraspanin CD151, but the role of these cells in immunity remains unknown [50]. Taken together, we identified a number of new downregulated genes in the current study, including *Pcdhga9*, *Cdc42ep5*, *Hrsp12*, *Rnasek*, *Nprl3*, *4932443I19Rik*, *Ly6g6c*, *Kdr*, *2810416G20Rik*, *Tubb2a*, *Triqk*, *Slc6a16*, *Cxx1c*, *Fez2*, *1810058I24Rik*, *Egfbp2*, *Efna5*, and *2210013O21Rik*. Whether these genes participate in the immune response needs to be investigated in the future.

The upregulated and downregulated genes in the current study are associated with 1672 and 1080 terms in the biological process, 137 or 134 terms in the cellular component, and 231 or 195 terms in the molecular function, indicating the importance of biological process in the regulatory mechanisms of *M. vaccae* vaccine. Interestingly, GO analysis demonstrated that the most significant GO term of upregulated genes in the biological process is the metabolic process. In contrast, the most significant GO term for downregulated genes in the biological process is localization. It is well known that the immune responses depend on energy. Maintaining adequate energy supply is the basis for immunocytes to attack *M. tuberculosis*. It has also been shown that *M. tuberculosis* can adhere to and taken up by alveolar epithelial cells [51, 52]. The interactions between *M. tuberculosis* and host molecules within the alveolar certainly play a key role in determining whether *M. tuberculosis* could successfully invade the host [53]. These findings suggested that *M. vaccae* vaccine activate more immunocytes to participate in the elimination of *M. tuberculosis* by enhancing metabolism, and antagonize the invasion of *M. tuberculosis* by downregulating the molecules involved in recognition, adhesion, and invasion.

KEGG pathway analysis in the current study identified 68 upregulated and 55 downregulated pathways by *M. vaccae* vaccination. The upregulated pathways most associated with *M. vaccae* vaccine treatment were TNF signaling pathway, NOD-like receptor signaling pathway, TLR signaling pathway, and mitogen-activated protein kinase (MAPK) signaling pathway. *M. tuberculosis* is primarily recognized by macrophages via TLR2/4 signaling pathways, but the TLR2 and TLR4 signal can be inhibited by the antigens secreted by bacteria [54], which makes it possible to inhibit autophagy, and allow the long-term presence of *M. tuberculosis* in macrophages [55]. After *M. vaccae* vaccination, the expression of TLR2 was significantly enhanced to induce upregulation of inflammatory cytokines (TNF-α, IL-6, IL-12, IL-18, and IL-1) and chemokines (CXCL, MCP-1) via MyD88-dependent TLR signaling pathway, NOD-like receptor signaling pathway, and subsequently activating two downstream pathways NF-κB and MAPK to accelerate the killing and elimination of *M. tuberculosis* [56–58]. MyD88 is one of the most extensively investigated adaptor proteins in the TLR signaling cascade, and plays a critical role in immune response to *M. tuberculosis* infection [59]. Our study determined that the expression of MyD88 is significantly upregulated in response to *M. vaccae* vaccine. However, MyD88-independent pathway also participates in the host defense against mycobacterial infection [57]. We speculate that *M. vaccae* vaccination could induce the transition of the TLR signaling pathway from MyD88-independent to MyD88-dependent.

Downregulated pathways associated with *M. vaccae* vaccination in the current study included focal adhesion, ECM-receptor interaction, Rap1 signaling pathway, and PI3K-Akt signaling pathway. Focal adhesions are integrin-containing, multi-protein structures that form mechanical links between intracellular actin bundles and the extracellular substrate in many cell types [60]. ECM is a highly dynamic structure that provides structural and biochemical support of surrounding cells [61, 62]. Both play a dominant role in the control of cell-cell and cell-matrix interactions by regulating the function of integrins and other adhesion molecules in various cell types. In addition, growth factor (GF) is a naturally occurring substance capable of stimulating cellular growth, proliferation, healing, and cellular differentiation [63]. In the present study, we found reduced expression of GF and ECM by the *M. vaccae* vaccine. Recognition of both molecules and their receptors on cell membrane could induce the activation of PI3K and FAK, thus triggering the downstream signaling events, including PI3K-Akt signaling pathway, Wnt signaling pathway, and Rap1 signaling pathway. These pathways have been implicated in macrophage invasion, *M. tuberculosis* survival, and impaired immune response [64, 65].

There are several limitations to this study. Firstly, the number of mice used to identify DE genes is relatively small (*n* = 3/group), and therefore must be considered preliminary. Secondly, the study was conducted in BALB/c mice; extrapolation to other animal species, and particularly human beings, must be cautious. Third, the changes induced by the *M. vaccae* vaccine were not compared to the BCG vaccine. Finally, the upregulated and downregulated signaling pathways were identified by bioinformatics based on microarray data; validation with more quantitative measures and at the protein levels is required.

## Conclusions

*M. vaccae* vaccine produces fairly robust protection against *M. tuberculosis*. The vaccination resulted in 2326 upregulated and 2221 downregulated genes and 68 upregulated and 55 downregulated pathways. Enhanced release of pro-inflammatory factors via MyD88-dependent TLR signaling pathway might be a key component of the action. Accelerated apoptosis of host cells due to downregulated PI3K-Akt signaling pathway could be another important mechanism.

## Supplementary information

**Additional file 1: Figure S1.** Array image of each sample. c1-c3, the serial number of mice in the control group; v1-v3, the serial number of mice in the *M. vaccae* group.

**Additional file 2: Table S1.** Raw and log2 value of the normalized intensity of each sample in the control group and the *M. vaccae* group.

**Additional file 3: Table S2.** Detail information of 2326 upregulated genes and 2221 downregulated genes.

**Additional file 4: Table S3.** GO analysis for upregulated DE genes in control and *M. vaccae* groups in terms of biological process (BP sheet), cellular component (CC sheet), and molecular function (MF sheet).

**Additional file 5: Table S4.** GO analysis for downregulated DE genes in control and *M. vaccae* groups in terms of biological process (BP sheet), cellular component (CC sheet), and molecular function (MF sheet).

**Additional file 6: Table S5.** The whole analysis results of upregulated pathways in control and *M. vaccae* groups.

**Additional file 7: Table S6.** The whole analysis results of downregulated pathways in control and *M. vaccae* groups.

## Data Availability

The datasets used during the current study are available from the corresponding author upon reasonable request.

## Abbreviations

AFP: Alpha-fetoprotein
BCG: Bacillus Calmette–Guérin
BP: Biological process
CC: Cellular component
CFDA: China Food and Drug Administration
CFUs: Colony formation units
DE: Differential expression
ECM: Extracellular matrix
GF: Growth factor
GO: Gene Ontology
HIV: Human immunodeficiency virus
IFN-γ: Interferon-gamma
IL: Interleukin
KEGG: Kyoto Encyclopedia of Genes and Genomes
M. tuberculosis: *Mycobacterium tuberculosis*
*M. vaccae*: *Mycobacterium vaccae*
MAPK: Mitogen-activated protein kinase
MDR: Multidrug-resistant
MF: Molecular function
NK: Natural killer
NOD: Nucleotide-binding oligomerization domain
PBMCs: Peripheral blood mononuclear cells
PI3K: Phosphatidylinositol-4,5-bisphosphate 3-kinase
PLA: People’s Liberation Army
PPD: Purified protein derivative
RELM/FIZZ: Resistin-like molecule/found in inflammatory zone
TB: Tuberculosis
Th-1: Helper T lymphocytes-1
TLR: Toll-like receptor
TNF-α: Tumor necrosis factor-alpha
WHO: World Health Organization
