# Supplementary figures and images for: Effects of Mycobacterium vaccae vaccine in a mouse model of tuberculosis: protective action and differentially expressed genes
Source: Mil Med Res. 2020 Jun 3;7:25. doi: 10.1186/s40779-020-00258-4 (PMC7268289; doi:10.1186/s40779-020-00258-4)

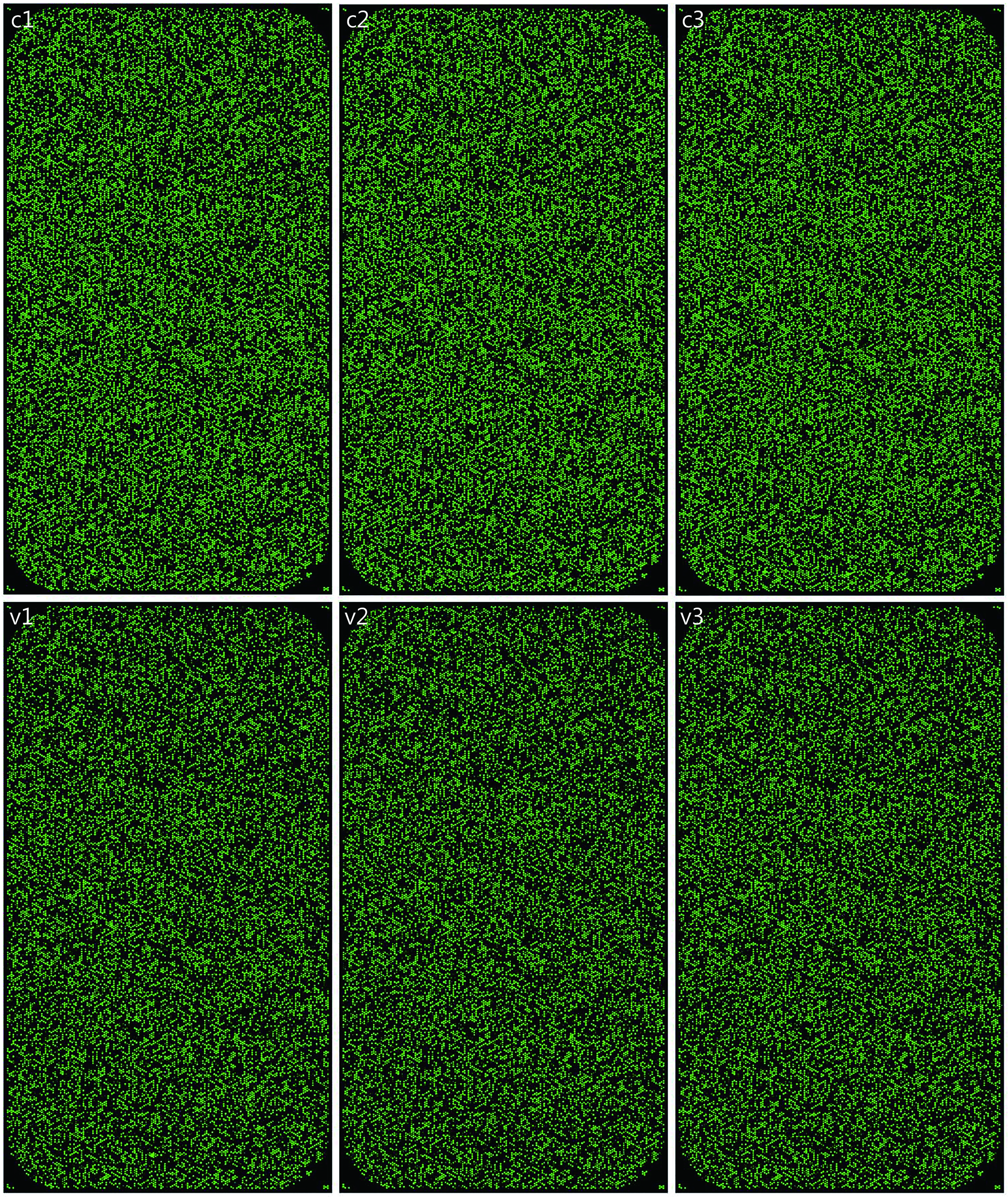

Supplement: Supplementary file 1 — Additional file 1: Figure S1. Array image of each sample. c1-c3, the serial number of mice in the control group; v1-v3, the serial number of mice in the M. vaccae group. [file 40779_2020_258_MOESM1_ESM.jpg]
